# Carotid to Left Subclavian Artery Bypass Grafting for the Treatment of Coronary Subclavian Steal Syndrome

**DOI:** 10.1016/j.cjco.2022.03.005

**Published:** 2022-03-19

**Authors:** Abdullah Baghaffar, Muhammed Mashat, Ryaan EL-Andari, Bruce Precious, Hashem Aliter, Christine Herman

**Affiliations:** a Division of Cardiac Surgery, Department of Surgery, Dalhousie University, Halifax, Nova Scotia, Canada; b Division of Cardiac Surgery, Department of Surgery, King Abdulaziz University, Jeddah, Saudi Arabia; c Faculty of Medicine and Dentistry, University of Alberta, Edmonton, Alberta, Canada; d Department of Diagnostic Imaging, Dalhousie University, Halifax, Nova Scotia, Canada

## Abstract

Recurrent angina after coronary artery bypass grafting is rarely caused by left subclavian artery (LSCA) stenosis resulting in reduced left internal mammary artery blood flow. We present 2 cases of coronary-subclavian artery steal syndrome resulting from LSCA stenosis and their successful surgical management with left carotid to LSCA bypass. Based on the successful management described in this case report, and the limitations of other options in addressing coronary-subclavian artery steal syndrome, left carotid to LSCA bypass surgery should be considered for revascularization in patients who develop postoperative coronary-subclavian artery steal syndrome due to LSCA stenosis.

## Résumé

La récidive d’angine après le pontage aortocoronarien est rarement causée par la sténose de l’artère sous-clavière gauche (ASCG) entraînant la réduction du débit sanguin de l’artère mammaire interne. Nous présentons deux cas de syndrome du vol coronaro-sous-clavier résultant de la sténose de l’ASCG et la réussite de leur prise en charge par pontage entre l’artère carotide gauche et l’ASCG. Compte tenu de la réussite de la prise en charge décrite dans cette observation et des limites des autres options dans le traitement du syndrome du vol coronaro-sous-clavier, le pontage entre l’artère carotide gauche et l’ASCG devrait être envisagé lors de la revascularisation des patients qui présentent le syndrome du vol coronaro-sous-clavier postopératoire en raison de la sténose de l’ASCG.

Coronary artery bypass grafting (CABG) surgery is an effective revascularization strategy utilized in treating coronary artery disease (CAD). The use of the left internal mammary artery (LIMA) to bypass the stenosed left anterior descending (LAD) coronary artery has become the gold standard, given its proven long-term patency and survival benefits.^1^ One potential complication of CABG is recurrent angina. Recurrent angina is most often caused by progressive stenosis in the graft or progression of native disease. A rare cause of recurrent angina is stenosis of the left subclavian artery (LSCA) resulting in recurrent ischemia secondary to reduced blood flow through the LIMA graft.^2^

Coronary-subclavian artery steal syndrome is an uncommon phenomenon, in which coronary flow is diverted into the LSCA through the patent LIMA conduit due to critical subclavian stenosis. Atherosclerotic disease of the proximal LSCA is the most common cause of coronary-subclavian artery steal syndrome. However, several other pathologic processes, such as Takayasu arteritis and radiation arteritis, can also compromise the subclavian artery flow.^2^ Herein, we present 2 cases of coronary-subclavian artery steal syndrome as a result of left subclavian artery stenosis with a history of CABG.

## Case 1

**Figure 1:**
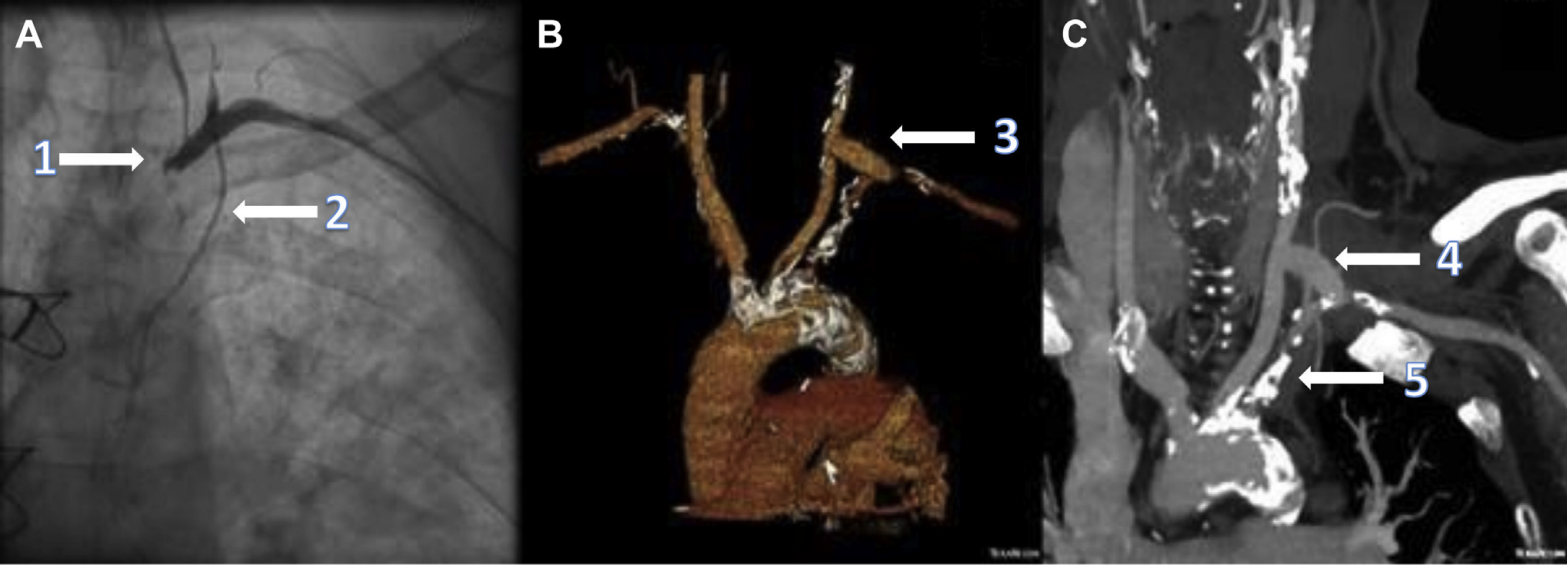
Cardiac catheterization, with an (**A**) anteroposterior view demonstrating complete occlusion of the (**1**) left subclavian artery (LSCA) and (**2**) patent left internal mammary artery graft, (**B**) postoperative cardiac computed tomography angiography with reconstruction showing the (**3**) newly implanted graft from the left carotid artery to LSCA, and (**C**) coronal view demonstrating the (**4**) patent graft from the left carotid artery to LSCA. Note the (**5**) extensive atherosclerotic disease in the origin of the LSCA.

A 71-year-old man with a history of CAD, hypertension, and diabetes previously underwent a 2-vessel CABG with LIMA to the LAD artery and a saphenous vein graft (SVG) to the obtuse marginal (OM) artery. He presented to the emergency department 2 years later, complaining of a 3-week history of shortness of breath at rest and chest tightness. He was admitted with a non-ST-elevation myocardial infarction and decompensated heart failure. An echocardiogram showed normal biventricular function with no regional wall motion or valvular abnormalities. Coronary angiography showed a patent SVG-to-OM graft and complete occlusion of the left subclavian artery (LSCA) causing steal syndrome of the patent LIMA graft (Fig. 1A).

Carotid Doppler showed no significant left carotid stenosis. The revascularization strategy chosen by the multidisciplinary team was a left carotid to subclavian artery bypass, which was uneventful. He was discharged 3 days postoperatively, with no evidence of ischemia.

## Case 2

A 70-year-old woman with a history of hypertension, dyslipidemia, diabetes, and CAD underwent a 4-vessel CABG with LIMA to LAD and SVG sequentially to the diagonal, OM, and posterior descending arteries. She presented to the emergency department nearly 10 years later, complaining of chest tightness and shortness of breath on exertion. She was admitted with a non-ST-elevation myocardial infarction and decompensated heart failure.

An echocardiogram showed an ejection fraction of 40%-45%, with moderate anterior and anteroseptal regional wall motion abnormality. Mild right ventricular systolic dysfunction was also present.

**Figure 2:**
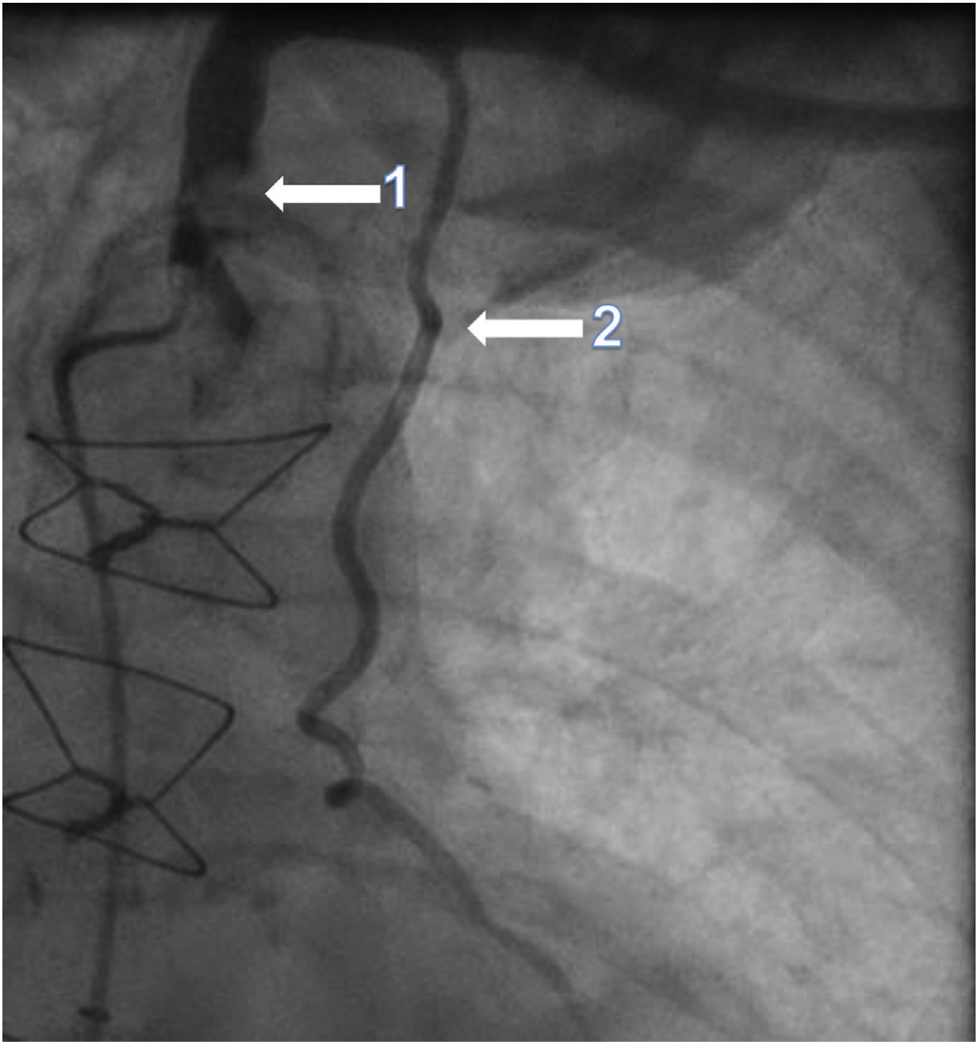
Cardiac catheterization, with an anteroposterior view demonstrating severe stenosis of the (**1**) left subclavian artery and (**2**) patent left internal mammary artery graft.

A coronary angiogram showed a widely patent LIMA graft to the LAD. However, the flow was compromised by a heavily calcified eccentric stenosis at the LSCA origin (Fig. 2). The sequential SVG to the diagonal, OM, and posterior descending arteries showed multifocal significant narrowings. Carotid doppler showed no significant disease. Retrograde flow was present within the left vertebral artery, in keeping with known LSCA disease.

The multidisciplinary team decided to proceed with a left carotid to subclavian artery bypass for coronary revascularization. She tolerated the procedure and was discharged the following day in a stable condition. The residual coronary stenoses were treated medically, as the postoperative myocardial perfusion scan showed no significant perfusion abnormalities.

## Operative Technique

**Figure 3:**
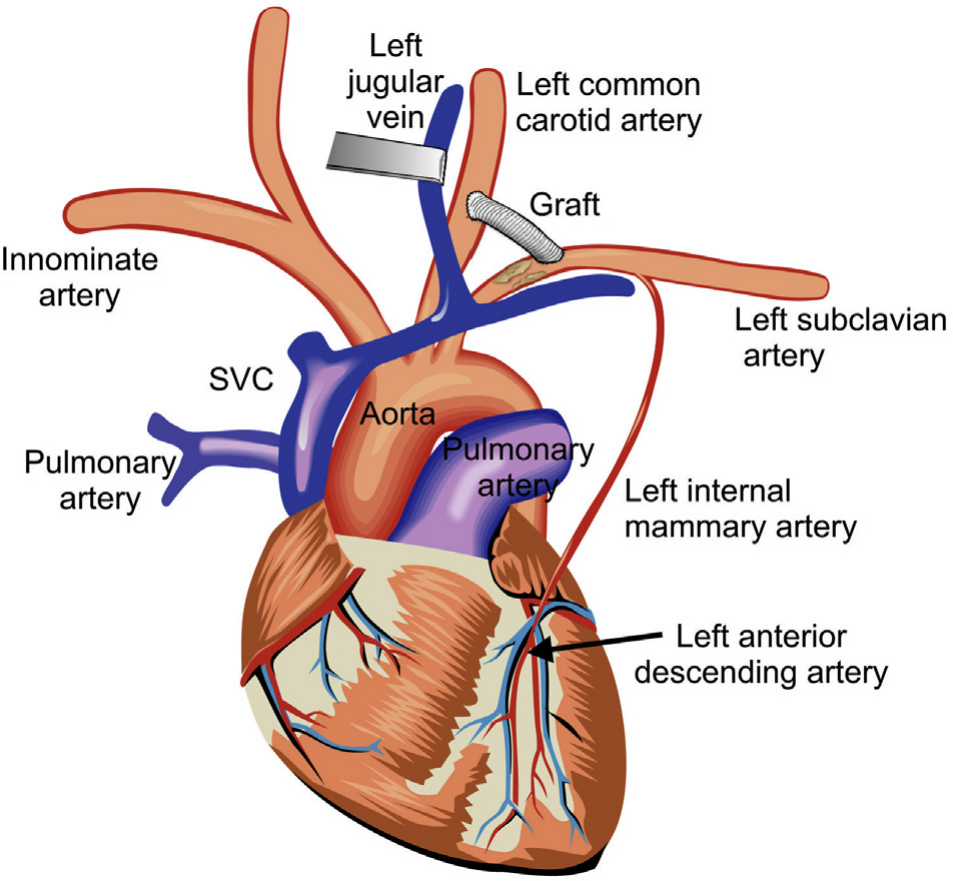
Diagram illustrating carotid to subclavian bypass graft distal to the stenotic segment of the left subclavian artery in a patient with previous coronary artery bypass grafting utilizing the left internal mammary artery graft to the left anterior descending coronary artery. SVC, superior vena cava.

Both procedures were performed in a similar fashion under general anesthesia. Near-infrared spectroscopy was utilized to monitor cerebral saturations intraoperatively. Through a left supraclavicular incision, the lateral head of the sternocleidomastoid was retracted medially. The scalene fat pad was excised to aid with exposure. The LSCA and the brachial plexus nerves were identified. The patients were then systemically heparinized. The LSCA was mobilized, achieving proximal and distal control. An 8-mm Dacron graft was anastomosed in an end-to-side fashion using 5-0 polypropylene suturing to the LSCA. It was then tunneled under the internal jugular vein (Fig. 3). The left common carotid artery was clamped proximally and distally, with no change in the near-infrared spectroscopy. The graft was anastomosed in an end-to-side fashion to the left common carotid artery, also using running 5-0 polypropylene suturing. Total clamp time of the carotid artery is approximately 10-15 minutes. Protamine was administered, and hemostasis was achieved. The incision was then closed in layers.

## Discussion

Recurrent angina is a concern post-CABG and may result in reduced quality of life and significant morbidity, especially if reoperation is required. Progression of LSCA stenosis has been found to cause ischemia, since the utilization of the LIMA for CABG has been widely adopted.^2^ Subclavian artery stenosis occurs in approximately 2% of the general population, 7% of those with peripheral arterial disease, and between 0.5% and 4% of people with CAD. Coronary-subclavian steal has been reported to complicate 0.2%-6.8% of CABG cases.^3^^,^^4^ Cases of coronary-subclavian steal in the early postoperative period are often due to preexisting stenosis of the subclavian artery or proximal LIMA.^3^, ^4^, ^5^ Risk factors for the development of late coronary-subclavian steal include atherosclerotic risk factors, such as diabetes, hyperlipidemia, hypertension, age, and tobacco abuse, along with less-common risk factors, such as Takayasu arteritis or stenosis secondary to radiation therapy.^3^ Signs of developing coronary-subclavian steal include recurrent angina without significant lesions in the coronary artery or LIMA, arm claudication, discrepant blood pressure between the arms, discrepant amplitude between the radial arteries, and carotid or subclavian bruits, which should be evaluated on preoperative physical exam.^3^, ^4^, ^5^ In these cases, the patients did not experience arm claudication or angina with the use of their left arm preoperatively or postoperatively.

Treatment options of coronary-subclavian artery steal syndrome include aorto-subclavian artery bypass, carotid-subclavian artery bypass, percutaneous interventions, and transposition of the LIMA.^4^^,^^6^ In our case, LSCA occlusion was treated with left carotid to LSCA bypass grafting (Fig. 1, B and C). In general, the risk of stroke is approximately 1%-2% for patients at this centre. Preoperatively, the contralateral carotid artery is assessed using carotid doppler ultrasound. Near-infrared spectroscopy is utilized to monitor cerebral saturations during all carotid interventions, and clamping is typically required for 10-15 minutes in order to perform the anastomosis.

Percutaneous transluminal angioplasty is an alternative for subclavian stenosis, and using the LIMA for coronary revascularization is a potential treatment in patients whose LSCA stenosis was diagnosed preoperatively. However, percutaneous transluminal angioplasty and stenting of the LSCA during the postoperative follow-up period may result in obstruction of the LIMA by the stent itself, transient ischemic attack or stroke, pseudoaneurysm formation, access-site hematoma, vessel rupture, dissection, stent migration, or distal embolization.^4^, ^5^, ^6^, ^7^ Additionally, percutaneous techniques are not always feasible. In some cases, such as chronic occlusions, proximal disease, and coronary steal, passage of a guidewire through the stenotic segment is difficult or impossible.^4^^,^^5^ Aorto-subclavian artery bypass or transposition of the LIMA maintains the advantage of the long-term patency of the LIMA. However, these strategies require a redo-sternotomy, which carries a higher risk of potential complications.^7^ In a meta-analysis comparing open vs endovascular treatment of subclavian artery stenosis, open techniques had improved rates of 1-, 3-, and 5-year patency over endovascular techniques. Rates of recurrent symptoms and survival did not differ significantly.^5^ But these were not patients with previous CABG. This difference in patency rate is of paramount importance in this population, as restenosis of the subclavian artery risks compromise of the LIMA graft, which may result in recurrent symptoms, reduced quality of life, risk of myocardial infarction, and mortality.

This case illustrates the successful use of carotid to subclavian bypass in addressing coronary-subclavian steal syndrome. Both patients had complete resolution of their symptoms immediately postoperatively. The patient in case 1 had recurrent shortness of breath with New York Heart Association class II symptoms 1 year postoperatively, but he did not have recurrent angina. Essential to the success of this procedure is a strong multidisciplinary team, to manage the various aspects of these complex cases. The multidisciplinary team involved in this case included those in vascular surgery, cardiac surgery, cardiology, anesthesiology, and radiology, all of which were essential in the planning and operative management of these cases.

Therefore, based on the successful management described in this case report, the limitations of other options in addressing coronary-subclavian artery steal syndrome, and previous literature demonstrating improved patency rates of the left subclavian artery following open vs endovascular repair, left carotid to LSCA bypass surgery should be considered for revascularization in patients who develop post-CABG coronary-subclavian artery steal syndrome due to LSCA stenosis. The decision for surgical or endovascular intervention should be made with a multidisciplinary team based on individual patient characteristics, such as symptoms, anatomy, and surgical risk.

**Novel Teaching Points**

• Left subclavian artery stenosis is a rare cause of recurrent angina after CABG.
• Carotid to subclavian bypass is an option to address coronary-subclavian steal syndrome.
• Carotid to subclavian bypass maintains long-term LIMA patency without requiring resternotomy.
